# Antibacterial efficacy of essential oil spray formulation for post-milking disinfection in dairy cows

**DOI:** 10.14202/vetworld.2023.1552-1561

**Published:** 2023-07-31

**Authors:** Jareerat Aiemsaard, Glenn Neville Borlace, Eakachai Thongkham, Chaiwat Jarassaeng

**Affiliations:** 1 Division of Pharmacology and Toxicology, Faculty of Veterinary Medicine, Khon Kaen University, Khon Kaen, 40002 Thailand; 2 Department of Pharmaceutical Chemistry, Faculty of Pharmaceutical Sciences, Khon Kaen University, Khon Kaen, 40002 Thailand; 3 Division of Theriogenology, Faculty of Veterinary Medicine, Khon Kaen University, Khon Kaen, 40002 Thailand

**Keywords:** antibacterial activity, essential oils, post-milking disinfection, spray formulation

## Abstract

**Background and Aim:**

Mastitis is an essential issue in dairy cows. Post-milking teat dips can help reduce this problem, but they employ harsh disinfectants, and many bacteria are becoming increasingly tolerant. This study aimed to investigate the antibacterial activity of clove, citronella, and sweet basil essential oils against the common bovine mastitis causative agents *Staphylococcus aureus*, *Streptococcus agalactiae*, and *Escherichia coli* and to develop an antiseptic post-milking teat spray for use in dairy cows.

**Materials and Methods:**

The *in vitro* antimicrobial activity of the essential oils was determined by broth microdilution and time-kill assays. Essential oil-based post-milking teat sprays were developed. The bacterial eradication efficacy of the formulations was determined by time-kill assays and their stability was tested by repeated freeze-thaw cycles. The most effective formulation was tested in dairy cows.

**Results:**

The minimum inhibitory concentrations and minimum bactericidal concentrations of the tested essential oils against *S. aureus*, *S. agalactiae*, and *E. coli* were in the range of 0.78–6.25 μL/mL. The time-kill tests indicated that the essential oils’ antibacterial activity depended on concentration and contact time. All three essential oil-based post-milking teat spray preparations showed good stability. The citronella spray formulation showed the highest antibacterial potency. In *in vivo* testing, the citronella spray eradicated aerobic bacteria on the teat skin of cows (99.9% or 3-log_10_ reduction) within 1 min, which was non-inferior to a standard 0.54% iodine solution teat dip.

**Conclusion:**

Clove, citronella, and sweet basil essential oils were effective against *S. aureus*, *S. agalactiae*, and *E. coli in vitro*. Of these, citronella essential oil is the most promising to be developed as a post-milking teat spray with high antibacterial activity and excellent bacterial eradication properties *in vivo*.

## Introduction

Bovine mastitis is an important disease in dairy cows worldwide that decreases milk quality and productivity and increases costs for treatment and herd health management. The most common cause is bacterial infection through cow-to-cow transmission of contagious bacteria such as *Staphylococcus aureus*, *Streptococcus agalactiae*, and *Streptococcus dysgalactiae* during milking or infection with environmental bacteria, such as *Escherichia coli* and *Streptococcus uberis* [1, 2]. There are several important risk factors for mastitis in dairy cows, including poor milking hygiene, especially not using post-milking disinfection, use of non-standard or deteriorated milking equipment, and improper management of the environment and herd health. Affected cows can develop sub-clinical or clinical mastitis depending on the virulence of the causative bacterium, the health of the cow, and the efficacy of herd management procedures [3].

Post-milking teat disinfection is the last step in the milking process. It decrease the number of microorganisms on the teat orifice and skin and reduce the chances of those microorganisms entering the teat cistern [4]. Several types of teat dip antiseptics are in use, including chlorhexidine, iodine compounds, and quaternary ammonium salts. The use of these compounds in a post-milking teat dip was shown to significantly reduce the incidence of clinical and sub-clinical mastitis in dairy cows and reduce the number of somatic cells and bacteria in raw milk compared with untreated cows [5]. However, using the same antiseptic for a long time, or using insufficient concentrations, may cause resistance in some bacteria and some reports have found cross-resistance between disinfectants and antibiotics due to increased excretion through efflux pumps [6, 7].

Essential oils contain many highly volatile phytochemical compounds, such as monoterpenes, sesquiterpenes, and phenylpropenes, with outstanding antioxidant and antimicrobial effects. Previous studies have revealed that clove, citronella, and sweet basil essential oils have excellent antimicrobial activity against both planktonic cells and biofilms of *Staphylococcus pseudintermedius* [8] and *S. aureus* [9, 10]. Thus, these essential oils may have the potential to be developed as antibacterial post-milking teat disinfection agents against the bacteria that cause mastitis.

This study aimed to investigate the antibacterial activity of clove, citronella, and sweet basil essential oils against the common bovine mastitis causative agents *S. aureus*, *S. agalactiae*, and *E. coli* and to develop an antiseptic post-milking teat spray for use in dairy cows.

## Materials and Methods

### Ethical approval

This study was approved by the Institutional Animal Care and Use Committee of Khon Kaen University, based on the Ethics of Animal Experimentation of National Research Council of Thailand (record no. IACUC-KKU-90/64).

### Study period and location

This study was conducted from June 2022 to January 2023 at the Faculty of Veterinary Medicine, Khon Kaen University, Thailand.

### Materials

The *S. agalactiae* was purchased from American Type Culture Collection (ATCC) 27956, Virginia, USA. The *S. aureus* was obtained from Department of Medical Sciences Thailand (DMST) 4745, Nonthaburi, Thailand. The *E. coli* was obtained from Thailand Institute of Scientific and Technological Research (TISTR) 527, Pathum Thani, Thailand. Clove, citronella, and sweet basil essential oils extracted by steam distillation were purchased from Thai-China Flavors and Fragrances Industry Co., Ltd., Ayutthaya, Thailand. The 0.54% iodine solution was from Evans Vanodine International PLC, Preston, UK. Mueller Hinton agar (MHA), Mueller Hinton broth (MHB), and Sabouraud dextrose agar (SDA) were purchased from Becton Dickinson, France, and dimethyl sulfoxide (DMSO) was purchased from V.S. Chem House, Ayutthaya, Thailand. All ingredients of the formulations were obtained from Chemipan Corporation Co., Ltd., Bangkok, Thailand.

### Bacterial culture and preparation

*Streptococcus agalactiae*, *S. aureus*, *and E. coli* were cultured in MHB at 37°C for 24 h. Inocula were prepared by diluting 24 h cultures with sterile phosphate-buffered saline (PBS) and bacterial concentration was determined by standard plate count [11].

### *In vitro* antimicrobial test of essential oils

#### Broth microdilution method

The assay was performed according to Clinical and Laboratory Standard Institute [11] guidelines with some modifications. Briefly, clove, citronella, and sweet basil essential oils were dissolved in DMSO to give a final concentration of 50 mg/mL. The stock essential oil solutions were then serially diluted 2-fold with MHB in 96-well round-bottomed microtiter plates (Corning Incorporated, USA). Plates were inoculated with 1 × 10^6^ colony-forming units (CFU)/mL bacteria per well. Wells containing only MHB and MHB with bacteria were used as negative and positive-growth control wells, respectively. After incubation at 37°C for 24 h, minimum inhibitory concentrations (MICs) were determined as the lowest concentration of the agent that inhibited visible growth. Then, 100 μL samples from the wells of MIC, 2× MIC, and 4× MIC were inoculated onto MHA and incubated at 37°C for 24 h. The minimum bactericidal concentrations (MBC) were determined from the lowest essential oil concentration that showed no MHA growth. Dimethyl sulfoxide was used as solvent control. All tests were performed in triplicate.

#### Time-kill tests

The time-kill test was conducted previously described by Aiemsaard *et al*. [12], with some modifications. Briefly, 900 μL of the diluted essential oil was thoroughly mixed with 100 μL of bacterial suspension to give final concentrations of 1, 5, 10, and 20× MIC. After incubation for 1, 15, 30, 60, and 180 min at 37°C, the mixture was 10-fold diluted with PBS to stop the reaction and 100 μL was spread onto MHA plates. After incubation at 37°C for 24 h, visible colonies were counted. Each experiment was performed in triplicate.

### Post-milking teat spray formulations

#### Formulation preparation

The spray-based ingredients are shown in Table-1 [13]. The essential oils were mixed with the spray-base at a final concentration that was shown to reduce the number of all three bacteria by at least 90% (1-log_10_ reduction) within 1 min, based on the time-kill test results. The final concentrations were 1% for clove essential oil and 3% for citronella and sweet basil essential oils.

**Table-1:** Ingredients of the post-milking teat spray-based formulation.

No.	Ingredient	Amount (%)
1	Sorbitan monooleate	0.5
2	Polyoxyethylene (80) sorbitan monooleate	5
3	Sodium dodecylbenzenesulfonate	1
4	Propylene glycol	10
5	Glycerin	5
6	Citrate-phosphate buffer pH 7^a^	Add to 100

a Citrate-phosphate buffer pH 7: 23.38 g of disodium hydrogen phosphate, 3.71 g of citric acid, and adjust pH to 7 using HCl or NaOH and add distilled water until volume is 1 L

#### Antimicrobial activity evaluation of the spray formulations

The antibacterial effects of the essential oil spray formulations were determined by the time-kill test [12]. Briefly, 900 μL of post-milking teat spray and 100 μL of bacterial suspension were mixed thoroughly. After incubation for 0.5, 1, 3, 5, and 10 min at 37°C, the mixture was 10-fold diluted with PBS to stop the reaction and 100 μL was spread onto MHA plates. After incubation at 37°C for 24 h, visible colonies were counted. Each experiment was performed in triplicate.

#### Microbial limits test

The essential oil sprays were tested for total aerobic bacteria and total yeasts and molds according to Thai Herbal Pharmacopoeia [14] with some modifications. Briefly, 1 mL of the formulation was diluted 10-fold with PBS to give 10^–1^–10^–3^ dilutions. Each dilution was inoculated onto MHA (bacterial test) and SDA plates (yeasts and molds test). The microbial colonies were counted after incubation at 25–30°C for 48 h. Each experiment was performed in triplicate.

#### Evaluation of physical properties and stability test

The color, sedimentation, and separation of the post-milking teat spray formulations were evaluated by visual observation. The pH was measured using a pH meter (Lab 850 set pH meter, SI Analytics, Germany) and viscosity was measured using a viscometer (Brookfield DVE viscometer, Brookfield Ametek, United States). Freeze-thaw stability testing was conducted by a method modified from de Villiers *et al*. [15]. Briefly, the sprays were subjected to six freeze-thaw cycles consisting of 24 h at −5°C followed by 24 h at 40°C before being assessed for their physical properties and in the time-kill assay and microbial limits test.

### *In vivo* antimicrobial test of post-milking teat spray

#### Animals

Fourteen Holstein Friesian cows with healthy udders (California mastitis test) that were producing milk and had not received any antibiotics for at least 21 days were included in the trial. The trial was conducted at a local dairy farm in Khon Kaen Province, Thailand, using a round-the-barn pipeline milking system.

#### Sample size calculation

The sample size was calculated for non-inferiority design with the data for a continuous variable by the program N4STUDIES version 1.4.1 (Chetta Ngamjarus, Nakhon Sri Thammarat, Thailand) according to the following equation [16]:


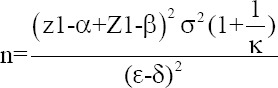


Where σ = standard deviation, ε = mean difference between groups (essential oil spray and 0.54% iodine solution), δ = non-inferiority margin, κ = ratio between groups, α = significance level (0.05), β = type II error probability (0.2), Z_1-α_ = 1.64, and Z_1-β_ = 0.84. As there are limited studies on the efficacy of essential oil-based post-milking teat disinfection, the variable values in the computational formula were predicted based on an efficacy study of teat dips conducted by Enger *et al*. [17]. Based on that study, the standard deviation of the percent bacterial reduction was 5%, the mean difference between groups was 10%, the non-inferiority margin was defined as a bacterial reduction equal to 20%, and the proportion between groups was 1:1.

#### Experimental design

This was a non-inferiority randomized controlled trial comparing the efficacy of the developed spray (Treatment 1) with 0.54% iodine teat dip (Treatment 2). The citronella essential oil formulation showed the highest antibacterial activity *in vitro* and was selected for the trial. One cow has four teats, so both treatments were randomly assigned to two teats on each cow to reduce the confounding bias. After the milking liners were removed, a 4 cm^2^ area of the skin of each teat was sampled using a sterile cotton swab which was then resuspended in 10 mL of PBS. The formulations were applied to the teat skin at a volume of 0.1 mL/1 cm^2^ for 1 min and then removed. Excess substances were removed by sterile gauze and teats were sampled using a sterile cotton swab which was then resuspended in 10 mL of PBS. Suspensions were inoculated onto MHA plates and incubated at 37°C for 24–48 h. The total aerobic bacteria was counted from visible colonies and compared between treatments [18, 19].

### Statistical analysis

The normality of the data was analyzed by the Shapiro–Wilk test. Differences in the total aerobic bacterial count before and after the application of each treatment and between treatments were assessed using Wilcoxon signed-ranks test (the data did not by the parametric statistical criteria). All tests were performed with a Statistical Package for the Social Sciences^®^ version 28 (IBM, Armonk, New York, United States).

## Results

### Broth microdilution assay

The antibacterial activities of the clove, citronella and sweet basil essential oils are shown in Table-2. The lowest MIC and MBC values were for clove essential oil (0.78–1.56 μL/mL). Citronella and sweet basil essential oils generated MIC and MBC values of 1.56–6.25 μL/mL.

**Table-2:** Antibacterial activity of clove, citronella, and sweet basil essential oil against *S. aureus* DMST 4745, *S. agalactiae* ATCC 27956, and *E. coli* TISTR 527.

Essential oil	Antibacterial activity (µL/mL)^a^					

	*S. aureus*		*S. agalactiae*		*E. coli*	

	MIC	MBC	MIC	MBC	MIC	MBC
Clove	0.78	0.78	0.78	1.56	0.78	0.78
Sweet basil	3.12	6.25	3.12	6.25	6.25	6.25
Citronella	3.12	6.25	1.56	1.56	6.25	6.25

a Values represent the minimum inhibitory concentration and the minimum bactericidal concentration collected from triplicate experiments. MIC=Minimum inhibitory concentration, MBC=Minimum bactericidal concentration, *S. aureus*=*Staphylococcus aureus*, *S. agalactiae*=*Streptococcus agalactiae*, *Escherichia coli*, DMST*=*Department of Medical Sciences Thailand, ATCC=American Type Culture Collection, TISTR=Thailand Institute of Scientific and Technological Research

### *In vitro* time-kill assay of essential oil

The time-kill study demonstrated that each essential oil generated a <1-log_10_ reduction in the number of tested bacteria at a concentration of 1× MIC after 180 min. At 5, 10, and 20× MIC, the essential oils showed more bactericidal activity, producing 3-log_10_ or 4-log_10_ reductions in bacterial numbers at several time points, indicating that the antibacterial effects of the essential oils were dose- and time-dependent.

**Figure-1:**
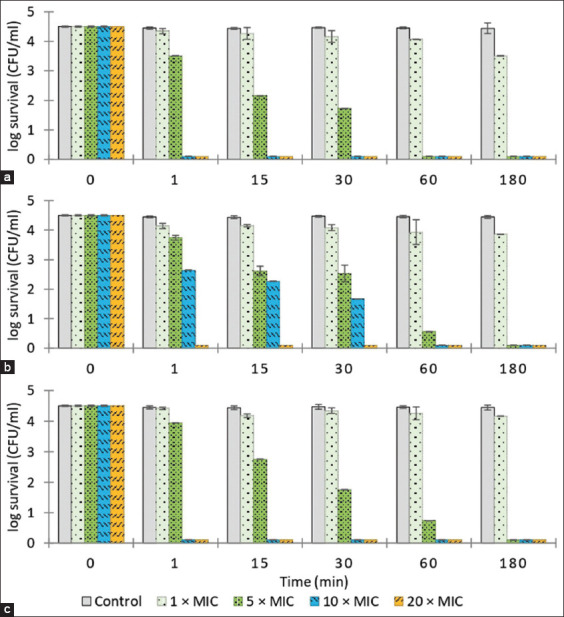
The time-kill assay of the essential oil against *Staphylococcus aureus*. (a) Clove essential oil; 1 × MIC = 0.78, 5 × MIC = 3.90, 10 × MIC = 7.80, and 20 × MIC = 15.60. (b) Citronella and (c) Sweet basil essential oil; 1 × MIC = 3.12, 5 × MIC = 15.60, 10 × MIC = 31.20, and 20 × MIC = 62.50. Control = PBS. MIC expressed in μL/mL. Values represent the mean of triplicate experiments with error bar (SD). MIC=Minimum inhibitory concentration, PBS=Phosphate-buffered saline, SD=Standard deviation.

**Figure-2:**
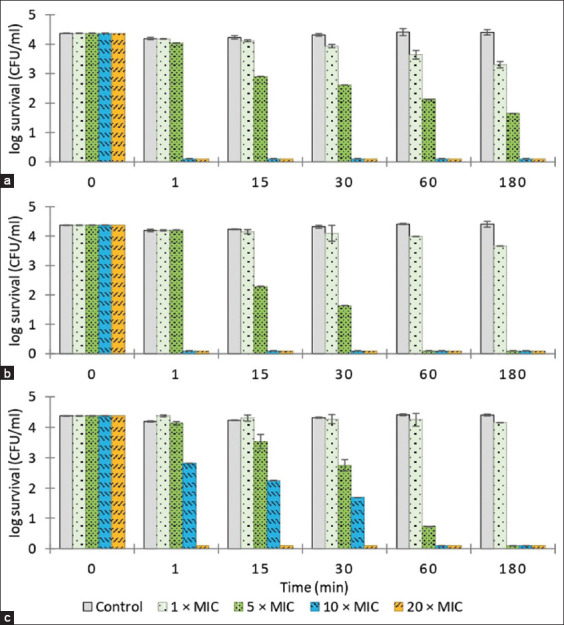
The time-kill assay of the essential oil against *Streptococcus agalactiae*. (a) Clove essential oil; 1 × MIC = 0.78, 5 × MIC = 3.90, 10 × MIC = 7.80, and 20 × MIC = 15.60. (b) Citronella essential oil; 1 × MIC = 1.56, 5 × MIC = 7.80, 10 × MIC = 15.60, and 20 × MIC = 31.20. (c) Sweet basil essential oil; 1 × MIC = 3.12, 5 × MIC = 15.60, 10 × MIC = 31.20, and 20 × MIC = 62.50. Control = PBS. Minimum inhibitory concentration expressed in μL/mL. Values represent the mean of triplicate experiments with error bar (SD). MIC=Minimum inhibitory concentration, PBS=Phosphate-buffered saline, SD=Standard deviation.

**Figure-3:**
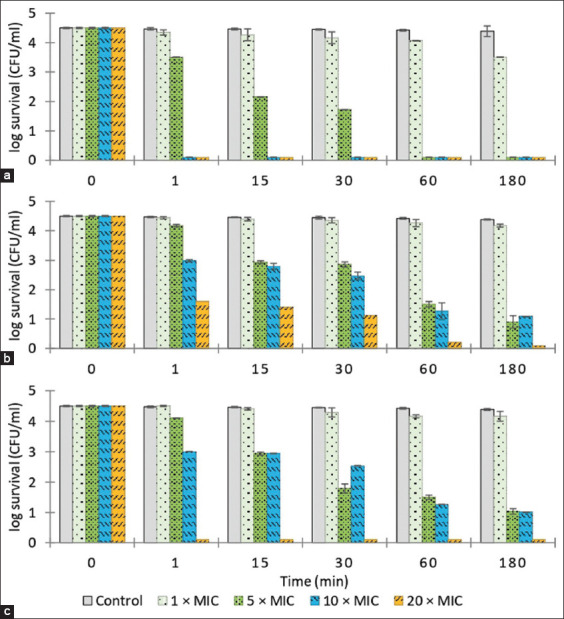
The time-kill assay of the essential oil against *Escherichia coli*. (a) Clove essential oil; 1 × MIC = 0.78, 5 × MIC = 3.90, 10 × MIC = 7.80, and 20 × MIC = 15.60. (b) Citronella and (c) sweet basil essential oil; 1 × MIC = 6.25, 5 × MIC = 31.25, 10 × MIC = 62.50, and 20 × MIC = 125.00. Control = PBS. Minimum inhibitory concentration expressed in μL/mL. Values represent the mean of triplicate experiments with error bar (SD). MIC=Minimum inhibitory concentration, PBS=Phosphate-buffered saline, SD=Standard deviation.

Figure-1 illustrates the time-kill kinetics of the essential oils against *S. aureus*. The clove and sweet basil essential oils at 10 and 20× MIC and citronella essential oil at 20× MIC eradicated *S. aureus* within 1 min (4-log_10_ or 99.99% reduction in CFU/mL) and all essential oils generated a 3-log_10_ or 4-log_10_ reduction in *S. aureus* CFU/mL after 60 min at 5× MIC. *Streptococcus agalactiae* was eradicated (4-log_10_ reduction in CFU/mL) within 1 min by essential oils of clove and citronella at 10 and 20× MIC and by sweet basil essential oil at 20× MIC. At 5× MIC, clove oil was not bactericidal after 180 min, inducing a <3-log_10_ reduction in bacterial numbers, but citronella and sweet basil produced 3-log_10_ or 4-log_10_ reductions in *S. agalactiae* CFU/mL after 60 min (Figure-2). Clove essential oil eliminated *E. coli* (4-log_10_ reduction in CFU/mL) after 1 min at 10 and 20× MIC and after 60 min at 5× MIC. Citronella essential oil at 20× MIC produced 3-log_10_ and 4-log_10_ reductions in *E. coli* CFU/mL after 15 min and 180 min, respectively, and caused a 3-log_10_ reduction in bacterial numbers after 60 min at 5 and 10× MIC. Sweet basil essential oil eliminated *E. coli* after 1 min at 20× MIC, and produced 3-log_10_ reductions in *E. coli* CFU/mL after 60 min at 5 and 10× MIC (Figure-3).

### Physical characteristics of essential oil post-milking teat sprays

All essential oil formulations had neutral pH (6.91–6.99) and low viscosity (44.50–85.00 cP) (Table-3). The clove essential oil spray appeared transparent, while the citronella and sweet basil oil sprays appeared cloudy. There were no significant differences in pH and viscosity values before and after the freeze-thaw cycles (p ≥ 0.05).

**Table-3:** pH and viscosity at 25°C of essential oil post-milking teat spray before and after FT.

Formulation	pH		Viscosity (cP)	
	
	Before FT	After FT	Before FT	After FT
1% v/v Clove oil	6.99 ± 0.00	6.97 ± 0.03	44.50 ± 0.71	22.00 ± 2.83
3% v/v Citronella	6.99 ± 0.01	6.98 ± 0.01	85.00 ± 1.41	64.00 ± 5.66
3% v/v Sweet basil	6.91 ± 0.01	6.88 ± 0.01	84.00 ± 2.83	39.00 ± 1.41

Values represent the mean ± SD of triplicate experiments. FT=Freeze-thaw test

### Microbial limit test

There was no visible growth of aerobic bacteria, yeasts, or molds for all three essential oil formulations after incubation on agar media for 48 h both before and after the freeze-thaw cycles (Table-4).

**Table-4:** Total aerobic bacterial count and total yeasts and molds count of essential oil post-milking teat spray before and after FT.

Formulation	Microbial limit test (CFU/g)			

	Total aerobic bacteria		Total yeasts and molds	
	
	Before FT	After FT	Before FT	After FT
1% v/v Clove oil	≤0.00	≤0.00	≤0.00	≤0.00
3% v/v Citronella	≤0.00	≤0.00	≤0.00	≤0.00
3% v/v Sweet basil	≤0.00	≤0.00	≤0.00	≤0.00

Values obtained from triplicate experiments. FT=Freeze-thaw test, CFU=Colony forming units

### *In vitro* time-kill assay of post-milking teat spray

**Figure-4:**
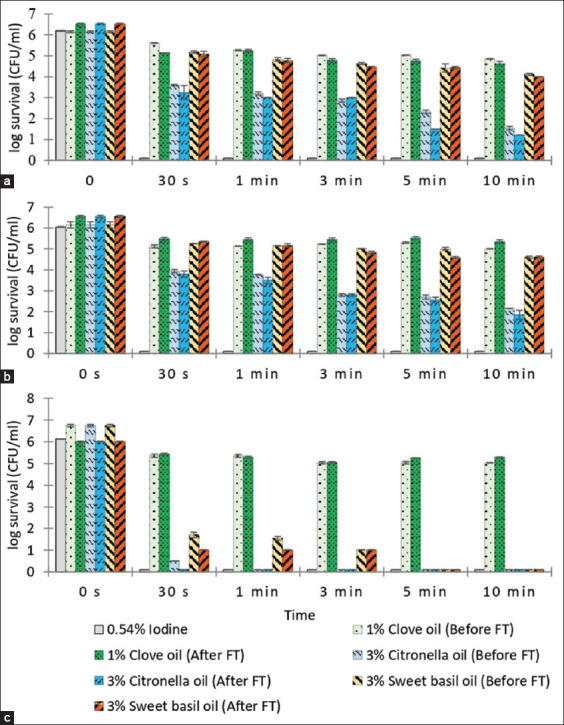
The time-kill assay of essential oil post-milking teat spray before and after freeze-thaw test (FT) and iodine post-milking teat dip formulation against (a) *Staphylococcus aureus*, (b) *Streptococcus agalactiae*, (c) *Escherichia coli*. Values represent means of triplicate experiments. The concentration expressed in % v/v except iodine expressed in % w/v.

The time-kill kinetics of the spray formulations are given in Figure-4. The 0.54% iodine solution (control) eradicated all three bacterial strains (6-log_10_ or 99.9999% reduction in CFU/mL) within 30 s. Citronella essential oil spray was the most bactericidal, decreasing the number of viable bacterial cells by 6-log_10_ (99.9999%) within 30 s for *E. coli* and causing >3-log_10_ reductions at 30 s (after freeze-thaw test [FT]) and 3 min (before FT) for *S. aureus* and 1 min (after FT) and 3 min (before FT) for *S. agalactiae*. The sweet basil essential oil spray showed strong bactericidal activity against *E. coli*, reducing the CFU/mL by 6-log_10_ within 30 s, but little activity against *S. aureus* and *S. agalactiae*, producing only 1-log_10_ to 2-log_10_ reductions in CFU/mL at 10 min. The clove essential oil spray showed little activity against all bacterial strains inducing only 1-log_10_ to 2-log_10_ reductions in bacterial numbers at 10 min. Freeze-thawing did not appear to affect the antibacterial properties of the essential oil formulations. The time-kill results for the freeze-thawed sprays were non-inferior to the sprays not subjected to freeze-thawing.

### *In vivo* bacterial eradication efficacy of post-milking teat spray

There were no significant differences (p ≥ 0.05) in the initial total aerobic bacterial cell counts on cow teat skin between the experimental group (sprayed with the citronella essential oil formulation) and the control group (dipped with the 0.54% iodine formulation). Both formulations showed a 100% reduction in the number of total aerobic bacteria 1 min after application (Table-5). Observation for the presence of any acute skin irritation showed no apparent pathologic lesions at 1, 5, and 10 min.

**Table-5:** Bacterial eradication efficacy of citronella and iodine formulations.

Formulation	Total aerobic bacterial cell count (CFU/cm^2^)		

	Before treatment	After treatment (1 min)	% Reduction
3% v/v Citronella teat spray	2.22 ± 6.49 × 10^3^	0.00 ± 0.00	100
0.54% w/v Iodine teat dip	3.36 ± 11.10 × 10^3^	0.00 ± 0.00	100

Values represent the mean ± SD of 14 cows, CFU=Colony forming units

## Discussion

The oils from clove, citronella, and sweet basil are inexpensive edible essential oils with good antimicrobial activity. This study demonstrated that all three essential oils had a good eradicating effect on the common bovine mastitis pathogens, *S. aureus*, *S. agalactiae*, and *E. coli*. The time-kill assays indicated that the antibacterial effects of these essential oils were dependent on both dose and time, which is consistent with a previous report by Aiemsaard *et al*. [20] about clove essential oil. Although there are limited studies of the time-kill kinetics of citronella and sweet basil essential oils, previous reports from our group have shown MIC values for citronella ranging from 0.25 to 4.4 μL/mL [8, 21] and from 0.125 to 6.25 μL/mL for sweet basil essential oil [8, 22], in accordance with the results of the present study.

The antibacterial activity of essential oils is believed to come from terpene (monoterpene and sesquiterpene) and phenolic (phenylpropene) chemical constituents such as citronellal, citronellol, and geraniol in citronella, chavicol in sweet basil, and eugenol in clove [21, 23]. These compounds damage the bacterial cell membrane, leading to increased membrane permeability and the leakage of intracellular substances to extracellular spaces [24, 25].

The post-milking teat sprays developed in this study are emulsion formulations since the active ingredients are lipophilic essential oils that cannot be readily dispersed in water. Using a mixed emulsifier system to make an emulsion ensures uniform droplet size and better stability [26, 27]. In the formulations developed here, polyoxyethylene (80) sorbitan monooleate, sodium dodecylbenzenesulfonate, and sorbitan monooleate were used as emulsifiers and combined with propylene glycol as a co-solvent. The finished products showed good stability with no deterioration in physical properties and antibacterial activity following six freeze-thaw cycles. In addition to these ingredients, we used glycerin as an emollient. Glycerin is an innocuous biocompatible compound that softens and moisturizes the teat skin and helps the formulation adhere to the skin [28]. In the formula design, we determined the concentration of essential oils based on the time-kill test results of each essential oil. The concentration of each oil capable of reducing the numbers of all three bacteria by at least 90% (1-log_10_ reduction) after 1 min was 1% v/v for clove essential oil and 3% v/v for the citronella and sweet basil essential oils. Although the *in vitro* broth microdilution and time-kill tests showed promising antibacterial effects from all three essential oils, only the sprays containing citronella and sweet basil essential oils retained their activity after being formulated with other ingredients.

The differences in the antimicrobial effect of essential oils before and after incorporation in the spray formulation may be due to the physicochemical properties of the active chemical constituents of the essential oil in the formula. The low concentration of clove essential oil employed in the clove spray formulation produced a transparent emulsion, which indicates a microemulsion. In contrast, the higher concentration of citronella and sweet basil essential oils in their spray formulations resulted in cloudy emulsions, which are indicative of macroemulsions. A previous study by Terjung *et al*. [29] reported that decreasing the droplet size of phenolic compounds such as eugenol and carvacrol in an emulsion increases sequestering in emulsion interfaces and decreases the concentration of these compounds in the aqueous phase, leading to reduced antimicrobial activity.

Spray formulations are convenient to use and have some advantages over conventional teat dips. First, the spraying device does not need to come in direct contact with the teats, which could help to reduce the transmission of infectious agents between animals. Second, using a spray ensures that the concentration of disinfectant is consistent across the area being sprayed. The developed citronella essential oil formulation reduced the number of *S. aureus* and *S. agalactiae* by 2-log_10_ to 3-log_10_ CFU/mL within 1 min and eliminated *E. coli* within 30 s in the *in vitro* experiments. The *in vivo* experiments confirmed that this spray retained this high antibacterial activity when applied to dairy cows in a farm setting and eliminated aerobic bacteria on the teat skin of cattle after application for 1 min.

## Conclusion

Clove, citronella, and sweet basil essential oils showed good *in vitro* antibacterial activity against three pathogens that commonly cause bovine mastitis: *Staphylococcus aureus*, *S. agalactiae*, and *E. coli*, with MIC and MBC values in the range of 0.78–6.25 μL/mL. When developed into post-milking teat sprays, it was found that citronella essential oil retained its bacterial eradication efficiency after incorporation into the spray formulation. *In vivo* experiments with dairy cows demonstrated that this formulation was effective against the bacterial fauna present on cow teat skin. This study suggests that citronella essential oil spray may be used as a post-milking teat antiseptic treatment for dairy cows and that the effect of essential oil spray on the incidence of mastitis in dairy cows and on milk quality should be further studied.

## Authors’ Contributions

JA and ET: Performed the *in vitro* antimicrobial testing of essential oils and post-milking teat spray formulations, statistical analysis, and drafted the manuscript. GNB: Designed the post-milking teat spray formulations, contributed to the manuscript drafting, and conducted a grammar review. CJ: Performed the *in vivo* antimicrobial test of post-milking teat spray and contributed to the manuscript drafting. All authors have read, reviewed, and approved the final manuscript.
